# Identification and Risk Assessment of Priority Control Organic Pollutants in Groundwater in the Junggar Basin in Xinjiang, P.R. China

**DOI:** 10.3390/ijerph20032051

**Published:** 2023-01-22

**Authors:** Zhi Tu, Yinzhu Zhou, Jinlong Zhou, Shuangbao Han, Jinwei Liu, Jiangtao Liu, Ying Sun, Fangyuan Yang

**Affiliations:** 1 College of Water Conservancy and Civil Engineering, Xinjiang Agricultural University, Urumqi 830052, China; 2 Xinjiang Hydrology and Water Resources Engineering Research Center, Urumqi 830052, China; 3 Xinjiang Key Laboratory of Hydraulic Engineering Security and Water Disasters Prevention, Urumqi 830052, China; 4 Center for Hydrogeology and Environmental Geology Survey, CGS, Baoding 071051, China; 5 College of Mathematics and Physics, Xinjiang Agricultural University, Urumqi 830052, China

**Keywords:** groundwater protection, the Junggar Basin, priority control organic pollutants, the *PvOPBT* method, ecological risk, health risk

## Abstract

The Junggar Basin in Xinjiang is located in the hinterland of Eurasia, where the groundwater is a significant resource and has important ecological functions. The introduction of harmful organic pollutants into groundwater from increasing human activities and rapid socioeconomic development may lead to groundwater pollution at various levels. Therefore, to develop an effective regulatory framework, establishing a list of priority control organic pollutants (PCOPs) is in urgent need. In this study, a method of ranking the priority of pollutants based on their prevalence (*Pv*), occurrence (*O*) and persistent bioaccumulative toxicity (*PBT*) has been developed. *PvOPBT* in the environment was applied in the screening of PCOPs among 34 organic pollutants and the risk assessment of screened PCOPs in groundwater in the Junggar Basin. The results show that the PCOPs in groundwater were benzo[a]pyrene, 1,2-dichloroethane, trichloromethane and DDT. Among the pollutants, benzo[a]pyrene, 1,2-dichloroethane and DDT showed high potential ecological risk, whilst trichloromethane represented low potential ecological risk. With the exception of benzo[a]pyrene, which had high potential health risks, the other screened PCOPs had low potential health risks. Unlike the scatter distribution of groundwater benzo[a]pyrene, the 1,2-dichloroethane and trichloromethane in groundwater were mainly concentrated in the central part of the southern margin and the northern margin of the Junggar Basin, while the DDT in groundwater was only distributed in Jinghe County (in the southwest) and Beitun City (in the north). Industrial and agricultural activities were the main controlling factors that affected the distribution of PCOPs.

## 1. Introduction

Groundwater is one of the most important water resources [1], and accounts for 68% of available freshwater resources in the world. More than 2.5 billion people worldwide rely on groundwater as their primary source of drinking water [2]. In China, socio-economic development and residents’ lives are heavily dependent on groundwater [3,4], which accounts for 30% of the total amount of water resources nationally [5]. Furthermore, groundwater is often used as the primary water supply source, sometimes even the only water supply source, in arid–semi-arid areas with scarce surface water resources [6,7,8]. In recent years, with socio-economic development, groundwater exploitation has increased significantly, with a widening variety of organic pollutants being transferred into groundwater [9,10,11]. Due to geogenic inferiority of groundwater and anthropogenic groundwater pollution, groundwater availability is restricted [12,13]. This exacerbates the water resource scarcity resulting from quality-induced water shortages in areas that heavily rely on groundwater as their main water supply source [14].

With the slow renewal rate [15] and complex aquifer structure of groundwater, once polluted, groundwater is extremely difficult to treat and restore [16]. However, the unified monitoring and management of all kinds of detected pollutants could be a huge drain on social resources. Therefore, the prioritized treatment of pollutants that have high detectable rates and pose great harm with limited resources is an effective pollution control strategy [17].

Many national governments and organizations published lists of priority control pollutants [18,19,20,21], which mainly focus on native surface water pollution status without regard for regional differentiation and groundwater pollution status. The hydrochemical characteristics of groundwater are strongly affected by the occurrence medium. Differences in hydrogeological conditions between various regions might also affect pollutant categories in groundwater [22]. When screening for priority control pollutants, therefore, regional disparity should be taken into consideration to devise a list of priority control pollutants and determine water supply safety. The common methods for screening priority control pollutants are high-performance liquid chromatography (HPLC) [23], the fuzzy comprehensive evaluation method [24,25], a calculation method based on the persistent bioaccumulative toxicity (*PBT*) of pollutants [26,27], the potential hazard index method [28,29] and the comprehensive ranking method [30,31]. Although the methods mentioned above have been widely applied in the identification of priority control pollutants, relevant studies are mainly focused on surface environmental mediums (e.g., surface water and atmosphere), and based only on the potential toxicity of pollutants to environmental biogroups without regard for local pollution status. Thus, the effects of these methods mentioned above are not ideal in practical applications on groundwater pollution [32]. On the other hand, a method of ranking priority pollutants based on their prevalence (*Pv*), occurrence (*O*) and *PBT* in the environment (the *PvOPBT* method) covers the shortages mentioned above, enabling one to compare the priorities of various pollutants and screen priority control pollutants in groundwater based on groundwater quality monitoring data, coupled with the toxicological characteristics and migration patterns of pollutants under given hydrogeological conditions [33].

The Junggar Basin in Xinjiang, located in the hinterland of Eurasia and away from the ocean, is a typical arid and semi-arid area with scarce rainfall, intense evaporation, an arid climate and a large portion of the Gobi Desert [2,34]. Groundwater, a significant resource, has important ecological functions and plays an important role in production and domestic water supply security in urban and rural areas, as well as supporting socio-economic development [35,36]. However, increasing anthropogenic activities during socio-economic development are often associated with groundwater pollution to a certain extent [37,38,39]. Since phreatic water with high vulnerability is widely distributed in the oasis zone in the Junggar Basin [40], groundwater pollution could represent a serious obstacle to sustainable development and urbanization construction if no timely prevention and control measures are offered [41,42]. Current studies of groundwater quality are mainly focused on the distribution, migration and transformation of inorganic components/pollutants, and there are few studies on organic pollution in this region. Thus, there is an urgent need to establish a list of priority control organic pollutants (PCOPs) to guide the development of relevant laws and regulations, and improve monitoring systems.

Therefore, the objectives of this study are to (1) identify PCOPs using the *PvOPBT* method, (2) carry out the ecological risk assessment and health risk assessment of PCOPs and (3) analyze the distribution and sources of PCOPs using the multi-analytical method.

## 2. Materials and Methods

### 2.1. Geological and Hydrogeological Conditions

#### 2.1.1. Geological Conditions

The Junggar Basin in Xinjiang is located in the northwest of China, with Altai Mountain to the north and Tianshan Mountain to the south, covering an area of 260 × 10^3^ km^2^ [40]. The topography of the basin is high in the east and low in the west, with an altitude of 600–700 m in the south, 800–1000 m in the northwest and 200–400 m in the west. Areas around the Ebinur Lake and the Manas Lake have the lowest altitude and are the main catchment area of surface water in the basin [43].

The plain area in the Junggar Basin, tectonically speaking, adopts the Caledonian fold as its basement, which has been continuously covered by younger sediments without folding during its geological history [44]. During the Paleogene–Neogene period, the Tianshan Mountain and the Altai Mountain uplifted along fractures, while the Junggar Basin subsided continuously, forming an intense depression zone with sediment thickness greater than 4000 m [43]. In the middle stage of the Quaternary period, alluvial–diluvial sediments were covered with loose fine soil with thickness ranging between tens to hundreds of meters, forming a spacious fine soil plain in front of the Tianshan Mountain. Moreover, the thickness of the Quaternary sediments decreases from the edge to the center. The Gurbantunggut Desert is located in the central basin, and the arid denudation high-plain (consisting of Tertiary and Paleozoic sediments) is located in the plain area on the north boundary of the Junggar Basin [15].

#### 2.1.2. Hydrogeological Conditions

**Figure 1:**
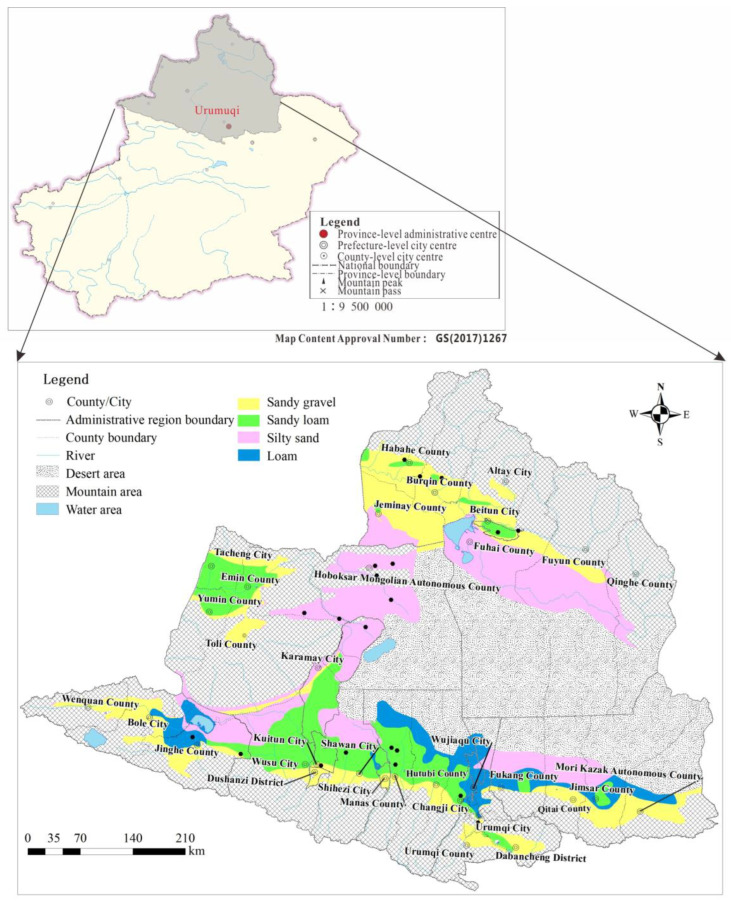
Lithology of vadose zone in the Junggar Basin.

The folds, faults, joints and fissures of the Tianshan Mountain, Altai Mountain and Junggar Mountain are well developed, and these provide advantageous conditions for the occurrence and migration of bedrock fissure groundwater. Groundwater in the plain area of the basin mainly occurrs in the Quaternary loose sediments of the piedmont alluvial–diluvial inclined plain and the lacustrine plain, which are the main areas of groundwater occurrence in the Junggar Basin [15,35]. The lithology of the vadose zone in the piedmont inclined plain is predominantly large-particle pebble and sand gravel, and that in the alluvial–diluvial plain below the spillover zone is dominated by finer sediment particles (sandy loam–silty–fine sand interlayer). The lithology of aquifers in the lacustrine plain in the south is predominantly silty–fine sand and loam (Figure 1).

The groundwater system in the Junggar Basin is relatively complete. There are abundant precipitation and widespread glaciers in the mountain area around the basin, which provide abundant water sources for groundwater formation. Therefore, it is regarded as a groundwater recharge area. With the intense incision of the ravines in the mountain area and frequent surface water–groundwater interactions, this area is regarded as a groundwater discharge area. Extra thick unconsolidated sediment in the plain area provides sufficient space for groundwater runoff and storage, and this is the main area of water resources utilization. The lacustrine basin area in the downstream region is the final groundwater discharge area. All the hydrogeological units mentioned above form a relatively complete groundwater system, with the Manas Lake, Ebinur Lake, Ulungur Lake and Zaysan Lake (in Mongolia) as the discharge areas [15]. Groundwater is naturally recharged by precipitation infiltration, river infiltration, hyporheic exchange in rivers and the lateral runoff of groundwater in mountain areas, and artificially recharged by canal seepage, field infiltration, reservoir water infiltration and the return infiltration of well irrigation water and spring water. Groundwater discharge generally involves artificial exploitation, spring water discharge, evapotranspiration and lateral runoff, etc. [45]. The hydrogeological conditions in the mountain area and plain area are quite different, resulting in different recharge–runoff–discharge conditions of the groundwater in various areas. Besides this, various basins in the plain areas have different recharge–runoff–discharge conditions due to the different hydrogeological conditions [15,43].

### 2.2. Sample Collection and Measurement

**Figure 2:**
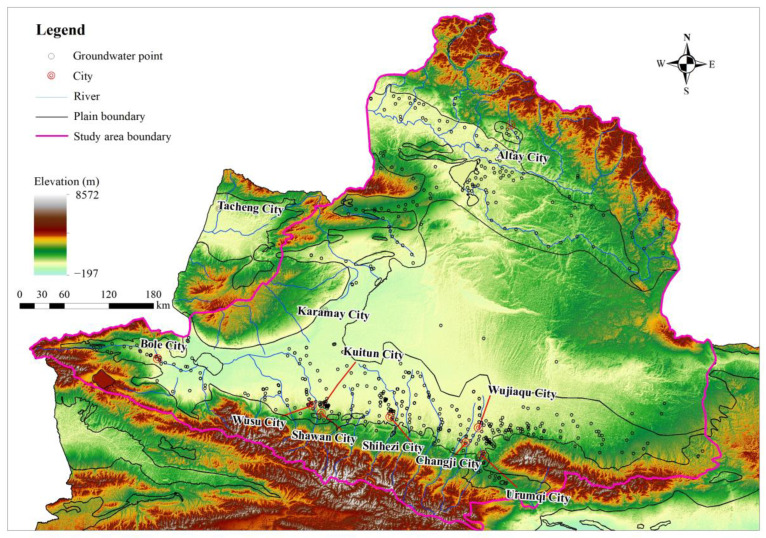
Distribution of groundwater sampling points in the study area.

With the exception of the Tacheng Basin, 455 groundwater samples were collected in the plain area of the Junggar Basin (Figure 2). All the groundwater samples were kept in a portable refrigerator and transported back to the lab at 4 °C to be analyzed within one week. According to investigation data on key pollution sources [46] and the Specification for regional groundwater contamination investigation and evaluation (DZT0288-2015) from China, a total of 34 organic pollutants were selected and tested (Table 1) at the Mineral Water Testing Center of Institute of Hydrogeology and Environmental Geology, Chinese Academy of Geological Sciences and National Research Center for Geo-analysis. The benzo[a]pyrene concentration was determined using the high-performance liquid chromatography (HPLC, Agilent1200) method, while other organic matters were tested using a gas chromatograph–mass spectrometer (GC-MS, Tracedsq GCMS-QP 2010, Thermo Fisher Scientific, Shanghai, China).

During groundwater collection, blanked samples and duplicate samples were collected to guarantee quality control. During groundwater analysis, both quality control and blanked samples were run every ten samples to check instrument stability and eliminate errors from the sampling procedures, respectively. The blanks were below the detection limits for all chemicals, and variations in the chemical concentrations of the quality control samples were within ±5%. The detections have a relative standard deviation (RSD) of ±10%. Certified reference materials (CRMs) were used for sample determination and all instruments used were calibrated and maintained weekly.

### 2.3. Screening Methods for PCOPs

The traditional *PBT* evaluation method that is applied in screening PCOPs focuses on pollutant accumulation in an environmental medium and potential toxicity to environmental biogroups, but it does not consider local pollution status. On the other hand, the optimized *PvOPBT* covers these shortages with a comprehensive consideration of groundwater organic pollution status and potential toxicity to the environmental medium. *Pv* and *O* represent the detectable rate and detection concentration of organic pollutants, respectively, which provide a direct basis for the assessment of the priority of organic pollutants. Therefore, PCOPs were assessed using the screening method based on a comprehensive score of *PBT*, *Pv* and *O*.

#### 2.3.1. Prevalence

The prevalence criterion (*Pv*) can be used to reflect the detection frequency of organic pollutant *i* in the study area to a certain extent (Equation (1)):

(1) $$Pv(i)=\frac{PD(i)}{N(i)}$$

where *PD*(*i*) is the quantity of detected organic pollutant *i*, and *N*(*i*) is the total quantity of tested organic pollutant *i*.

#### 2.3.2. Occurrence

The occurrence *O*(*i*) includes *O*_1_(*i*) and *O*_2_(*i*), which reflect the highest environmental concentration of organic pollutant *i* and the proportion of high environmental concentration of organic pollutant *i*, respectively. To eliminate the extremum effect, *O*_1_(*i*) is taken as the 95th percentile of the logarithm of organic pollutant concentration when *PD*(*i*) >3. When *PD*(*i*) ≤ 3, *O*_1_(*i*) is taken as the minimum of the 95th percentile of the logarithm of organic pollutant concentration (Equation (2)). This process compensates for the lack of statistical significance of small datasets and reduces calculation errors caused by limited data volumes [33].

(2) $$O_{1}(i)=\left\{\begin{array}{cc} \left(\log{EC}_{95\mathrm{th}}\right)(i) & \left(PD(i)>3\right)\\ \min\left(\left(\log{EC}_{95\mathrm{th}}\right),(,i,)\right) & \left(PD(i)\leq 3\right) \end{array}\right\}$$

where *EC*_95th_ is the 95th percentile of the logarithm of the environmental concentration of organic pollutant *i*.

*O*_2_(*i*) represents for the proportion of environmental concentration that exceeds the optimized predicted non-effect concentration (*PNEC_f_*(*i*)). When the organic pollutant concentration > *PNEC_f_*(*i*) (i.e., high environmental concentration), it is considered to have a detrimental impact on ecosystem structure and function [47]. The *PNEC_f_*(*i*) value was calculated based on the predicted non-effect concentration (*PENC*) and conservative factor *F*(*i*) (Equations (3) and (4)). Since the *PNEC*(*i*) value might be greater than the *EC*(*i*) value in many cases, optimization should be carried out by coupling with conservative factor *F*.

(3) $$PNEC_{f}(i)=\frac{PNEC(i)}{F(i)}$$

(4) $$F(i)=\frac{median(PNEC(i))}{median(EC(i))}$$

where *PNEC* is calculated based on the concentration required to see 50% of the maximal effect (*EC*50) or the lethal concentration 50 (*LC*50) of the toxicity of organic pollutant *i* at three trophic levels (fish, daphnia and algae) (Equation (5)).

(5) $$PNEC(i)=\frac{\min(EC50\mathrm{or}LC50)}{\mathrm{AF}}$$

where the *EC*50 or *LC*50 toxicity data (fish, daphnia and algae) of organic pollutant *i* are calculated using the ecological structure–activity relationship model (ECOSAR, EPI Suite) [48]. Assessment factor (AF) is set to 1000 [28].

When *PD*(*i*) ≤ 3, the *O*_2_(*i*) value (Equation (6)) is set to 0. This process could eliminate the calculation errors that arise in small datasets or data without significant differences.

(6) $$O_{2}(i)=\left\{\begin{array}{cc} \frac{{PD}_{EC>PNECf(i)}}{PD(i)} & \left(PD(i)>3\right)\\ 0 & \left(PD(i)\leq 3\right) \end{array}\right\}$$

where *PD_EC_*_> *PNECf*(*i*)_ is the amount of detected organic pollutants with concentrations greater than the *PNEC_f_* (*i*).

The *O*(*i*) is calculated via the combination of *O*_1_(*i*) and *O*_2_(*i*) (Equation (7)).

(7) $$O(i)=\frac{1}{2}\times (U(O_{1}(i))+U(O_{2}(i)))$$

where *U* is the utility function used to linearly convert each sub-criteria to a value between 0 and 1.

#### 2.3.3. PBT

The *PBT*, which consists of persistence, bioaccumulation and toxicity factors, is calculated based on previous methods using three parameters acquired from the QSAR modeling software EPI Suite (EPA, 2011) [26,49]. The persistence of organic pollutant *i* (*P*(*i*)) is acquired using the ultimate biodegradation value (Biowin3(*i*)) in the BioWin model coupled with Equation (8) [50]. The bioaccumulation of organic pollutant *i* (*B*(*i*)) is acquired using the bioaccumulation factor (*BCF*(*i*)) calculated from the BCFBAF model coupled with Equation (9) [51]. The toxicity of organic pollutant *i* (*T*(*i*)) is obtained using the LC50(*i*) value for fish calculated from the EcoSAR model coupled with Equation (10) [49]. The *PBT*(*i*) is calculated from the weighted mean of *P*(*i*), *B*(*i*), and *T*(*i*) (Equation (11)).

(8) P(i)=U(−Biowin3(i))

(9) B(i)=U(logBCF(i))

(10) $$T(i)=U\left(\frac{1}{LC50(i)}\right)$$

(11) $$PBT(i)=\frac{1}{3}\times (U(P(i))+U(B(i))+U(T(i)))$$

#### 2.3.4. PvOPBT Scores

The final score of *PvOPBT*(*i*) is calculated from the combination of *Pv*(*i*), *O*(*i*) and *PBT*(*i*) (Equation (12)).

(12) $$PvOPBT(i)=\frac{1}{3}\times (U(Pv(i))+U(O(i))+U(PBT(i)))$$

Organic pollutants in the high-priority category are identified as priority control pollutants based on *PvOPBT*(*i*) score classification using the K-means clustering method.

### 2.4. Risk Assessment Methods

#### 2.4.1. Ecological Risk Assessment

The ecological risk of PCOPs is assessed based on the hazard quotient (HQ) value (Equation (13)) [52]

(13) $$HQ(ij)=\frac{EC(ij)}{PNEC(i)}$$

where *EC*(*ij*) is the measured concentration of pollutant *i* collected at sampling point *j*. the *PNEC*(*i*) value is calculated from Equation (5). The *RQ* values, reflecting the degree of ecological risk, can be categorized into four levels including no risk (*HQ* < 0.01), low risk (0.01 ≤ *HQ* < 0.1), medium risk (0.1 ≤ *HQ* < 1) and high risk (*HQ* ≥ 1) [53].

#### 2.4.2. Health Risk Assessment

According to the Human Health Evaluation Manual [54], potential human health risk *R*(*ij*) is the value at risk of pollutants hazardous to human health, which is mainly influenced by average chronic daily intake (*CDI*) and the slope factor (*SF*) (Equations (14) and (15)). The upper limit and generally acceptable level of *R*(*ij*) are 1 × 10^−4^ and 1 × 10^−6^, respectively [54,55].

(14) R(ij)=CDI(ij)×SF(i)

(15) $$CDI(ij)=\frac{EC(ij)\times \mathrm{IR}\times \mathrm{EF}\times \mathrm{ED}}{\mathrm{BW}\times \mathrm{AT}}$$

where *CDI*(*ij*) represents the chronic daily intake dose (mg·(kg·d)^−1^); *SF*(*i*) is the result of a low-dose extrapolation procedure and is presented as the carcinogenic risk (mg·(kg·d^−1^)) [56]. IR is the intake rate (L·d^−1^) (the IR values for a child and an adult are set to 1 and 2, respectively); EF is exposure frequency (d·a^−1^); ED is exposure duration (a) (ED values for a child and an adult are set to 6 and 70, respectively); BW is body weight (kg) (BW for child and adult are set to 14 and 60, respectively); AT is average exposure time (d) (AT for child and adult are set to 2190 and 25,550, respectively) [57].

## 3. Results

### 3.1. Evaluation Scores of Organic Pollutants

**Figure 3:**
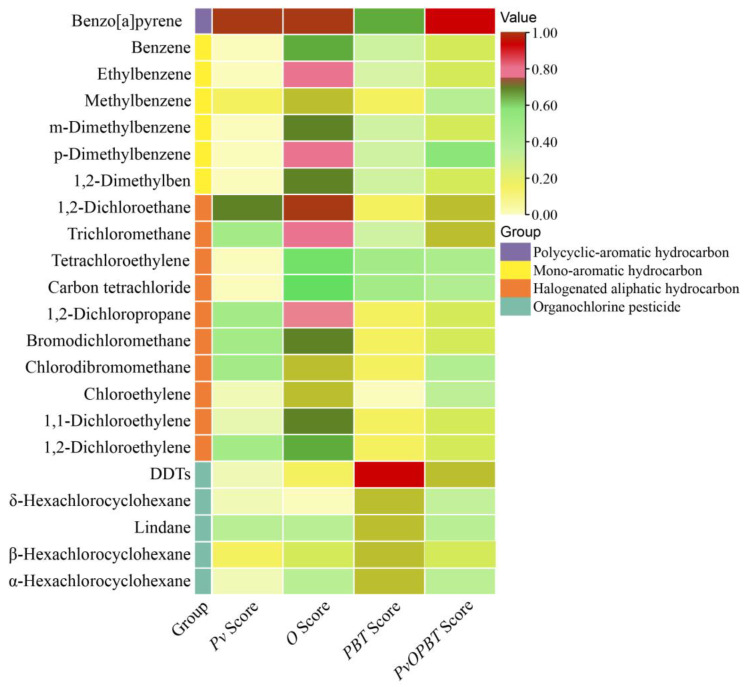
Classification and evaluation scores of organic pollutants in groundwater.

Among the 34 tested organic pollutants, there were 12 non-detected organic pollutants and 22 detected organic pollutants (including polycyclic-aromatic hydrocarbon, mono-aromatics hydrocarbon, halogenated aliphatic hydrocarbon and organochlorine pesticide) (Figure 3). In the polycyclic-aromatic hydrocarbons group, only benzo[a]pyrene was detected, but this had the highest *Pv* score, *O* score and *PvOPBT* score among all 22 of the detected organic pollutants. In the mono-aromatics hydrocarbon group, methylbenzene had the highest *Pv* score, p-dimethylbenzene had the second highest *O* score and *PvOPBT* score, and benzene had a relatively high *PBT* score. In the halogenated aliphatic hydrocarbon group, 1,2-dichloroethane had the highest *Pv* score, *O* score and *PvOPBT* score, and tetrachloroethylene had the second highest *PBT* score. In the organochlorine pesticide group, β-hexachlorocyclohexane had the highest *Pv* score and *O* score, and DDTs had the highest *PvOPBT* score. Among all 22 of the detected organic pollutants, DDTs had the highest *PBT* score.

Among all four organic pollutant groups, the polycyclic-aromatic hydrocarbon group had the highest *Pv* score, whilst the mono-aromatics hydrocarbon group had the lowest *Pv* score. In terms of *O* score, the polycyclic-aromatic hydrocarbon group and mono-aromatics hydrocarbon group were generally high, while the halogenated aliphatic hydrocarbon group showed the largest score range (0.37–0.93). As regards the *PBT* score, the polycyclic-aromatic hydrocarbon group and organochlorine pesticide had relatively high scores, while mono-aromatics hydrocarbon and halogenated aliphatic hydrocarbon had relatively low scores. Since the *PBT* score was calculated using the QSAR and ECOSAR models, there was a small difference in the *PBT* score among pollutants with similar compound structures. The final *PvOPBT* score, the assessment basis for identifying PCOPs, was much higher in the polycyclic-aromatic hydrocarbon group than in the other three groups. However, the *PvOPBT* scores differed little among the mono-aromatics hydrocarbon, halogenated aliphatic hydrocarbon and organochlorine pesticide groups.

### 3.2. Priority Ranking of Organic Pollutant Categories and Scores of Priority Organic Pollutants

**Figure 4:**
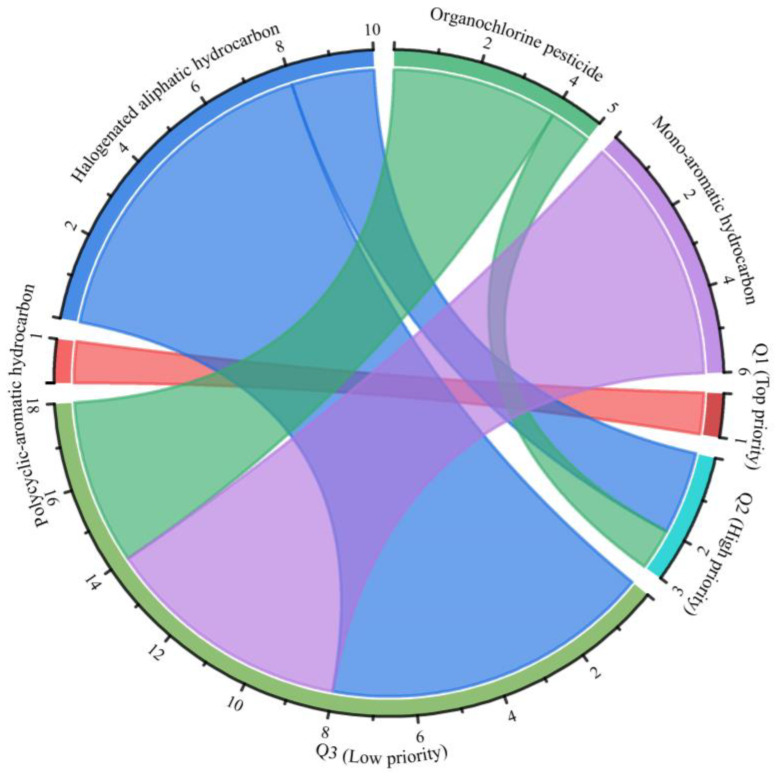
Proportion and quantity of priority levels for various categories of groundwater organic pollutants.

The *PvOPBT* scores of organic pollutants were ordered and categorized into three priority levels (i.e., top priority, high priority and low priority) using the K-means clustering method (Figure 4). Among all the organic pollutants, only the polycyclic-aromatic hydrocarbon was identified as a top priority. In total, 13.6% of the organic pollutants were identified as high priority, of which 66.7% were halogenated aliphatic hydrocarbons and 33.3% were organochlorine pesticides. The majority of organic pollutants (81.8%) were identified as low priority, of which 33.3% were mono-aromatics hydrocarbons, 44.5% were halogenated aliphatic hydrocarbons and 22.2% were organochlorine pesticides. Organic pollutants of top and high priority generally had high comprehensive scores (*Pv*, *O*, *PBT*), indicating a high level of hazard to human health and the environment. Therefore, the PvOPBT ranking scores that placed organic pollutants in the top and high priority groups were identified as PCOPs. The major groundwater PCOPs were polycyclic-aromatic hydrocarbon, halogenated aliphatic hydrocarbon and organochlorine pesticide in the Junggar Basin.

**Figure 5:**
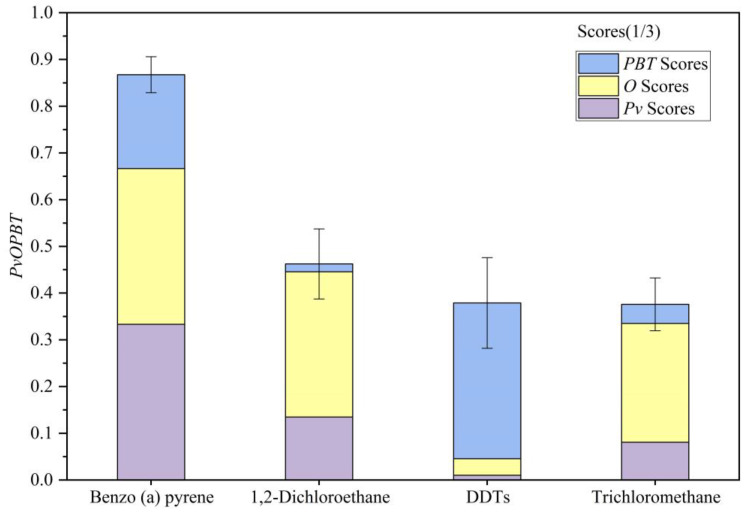
*PvOPBT* scores for PCOPs in groundwater.

The compositions of the *PvOPBT* ranking scores for PCOPs are presented in Figure 5. The mean of the *Pv* score, the *O* score and the *PBT* score of PCOPs were 0.14, 0.23 and 0.15, respectively, suggesting that high priority organic pollutants were determined by their concentration. The opposite was true for DDTs, the high control priority of which was determined by their high *PBT* score (with low *Pv* score and *O* score).

### 3.3. Spatial Distribution of PCOPs in Groundwater

**Figure 6:**
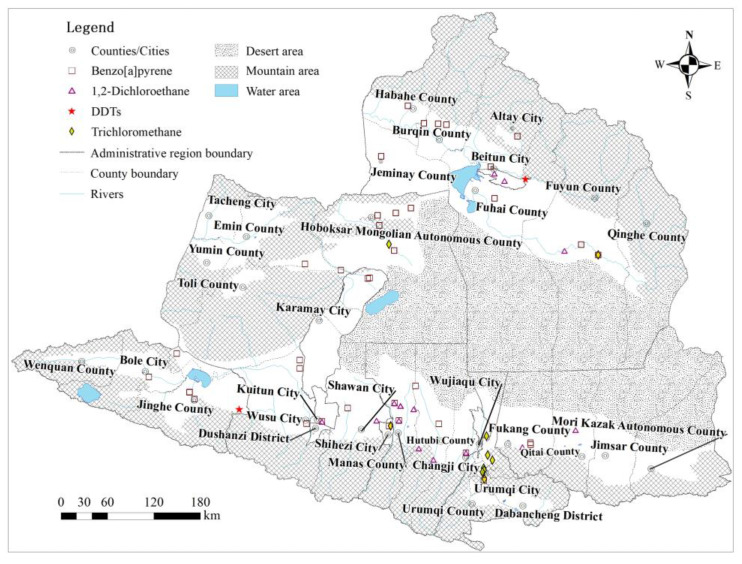
Distribution of PCOP in groundwater.

All the kinds of PCOPs in the groundwater were distributed in the southern margin of the Junggar Basin (Figure 6), and all the DDTs were distributed in Jinghe County and Beitun City, while the high levels of trichloromethane (80%), 1,2-dichloroethane (79%) and benzo[a]pyrene (32%) detected in groundwater were mainly concentrated in the central part. Moreover, large amounts of trichloromethane (70%) were distributed in Urumqi City, where the benzo[a]pyrene concentration in the groundwater was much higher than in other areas.

In the central Junggar Basin, benzo[a]pyrene was distributed in the Karamay City and Hoboksar Mongol Autonomous County, and trichloromethane was distributed in the Hoboksar Mongol Autonomous County (Figure 6).

In the northern Junggar Basin (with the exception of Qinghe County), benzo[a]pyrene was distributed in all the cities/counties. Besides this, the 1,2-dichloroethane and DDTs were distributed in Beitun City, and 1,2-dichloroethane and trichloromethane were distributed in Fuyun County (Figure 6).

### 3.4. Results of Risk Assessment

#### 3.4.1. Ecological Risk Assessment

**Figure 7:**
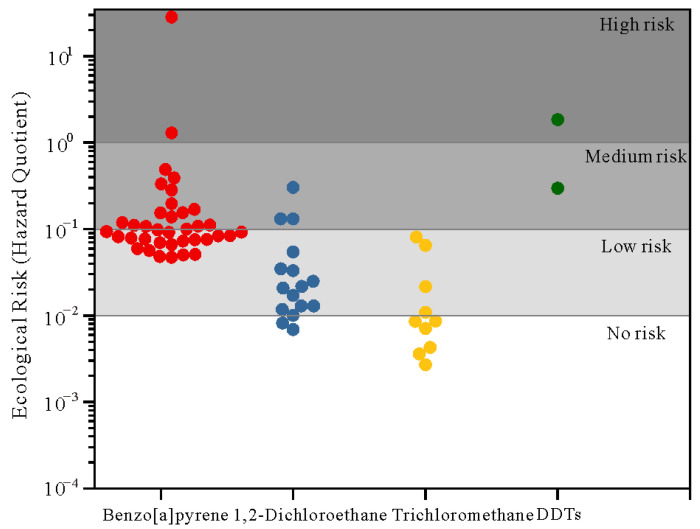
Ecological risk of PCOPs in groundwater.

The results of the ecological risk assessment (Figure 7) show that 66.7% of the detected PCOPs represented no to low ecological risk, and that the levels of trichloromethane detected in all samples posed no to low ecological risk. In terms of the organic pollutants posing medium ecological risk, benzo[a]pyrene accounted for the highest proportion (78.9%), followed by 1,2-dichloroethane (15.8%) and DDTs (5.3%). Only 5% of benzo[a]pyrene and 50% of DDTs posed a high ecological risk.

#### 3.4.2. Health Risk Assessment

**Figure 8:**
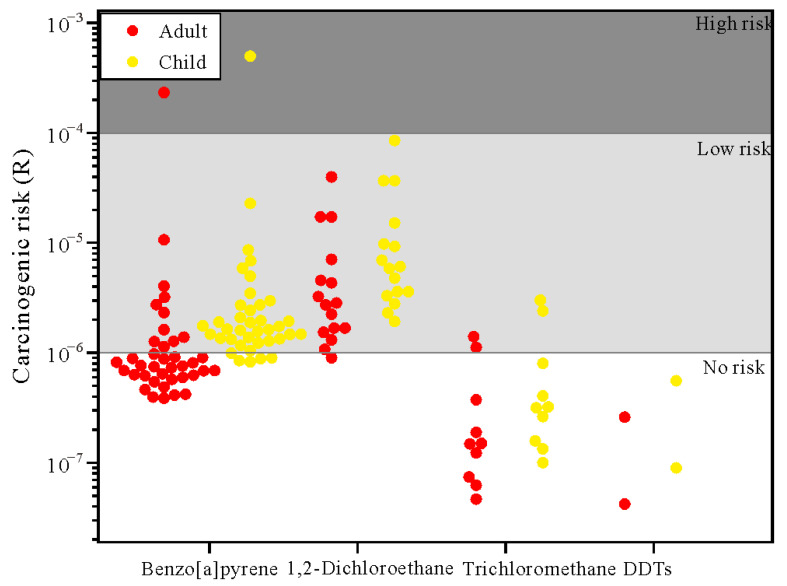
Health risk of PCOPs in groundwater.

The mean carcinogenic risk scores of benzo[a]pyrene, 1,2-dichloroethane, trichloromethane and DDTs for adult were 7.39 × 10^−6^, 6.84 × 10^−6^, 3.69 × 10^−7^ and 1.51 × 10^−7^, respectively, and those for children were 1.59 × 10^−5^, 1.46 × 10^−5^, 7.92 × 10^−7^ and 3.25 × 10^−7^, respectively. Among these, the mean carcinogenic risk score of PCOPs for children was 2.15 times of that for adults. A health risk assessment (Figure 8) suggested that 40.2% of the detected PCOPs posed no carcinogenic risk, 58.3% of the detected PCOPs posed low carcinogenic risk, and 1.5% of the detected PCOPs posed high carcinogenic risk. The potential carcinogenic risk scores of PCOPs in descending order were benzo[a]pyrene, 1,2-dichloroethane, trichloromethane and DDTs. Besides this, the risk for children was found to be higher than that for adults.

## 4. Discussion

In this study, PCOPs were identified using the *PvOPBT* method with a comprehensive consideration of the pollutant’s toxicity and its accumulation in the environmental medium based on the groundwater organic pollution status. Benzo[a]pyrene, 1,2-dichloroethane, trichloromethane and DDTs were identified as the PCOPs in the groundwater in the Junggar Basin, and these feature on the list of PCOPs established by the China National Environmental Monitoring Centre [19] and the US EPA [18].

### 4.1. Benzo[a]pyrene

Benzo[a]pyrene, which is poorly soluble in water and difficult to biodegrade, is the most toxic carcinogen of the polycyclic-aromatic hydrocarbons (PHAs) [58,59]. It can be derived from a wide range of sources via natural processes and anthropogenic activities. However, the amount and concentration of benzo[a]pyrene produced via natural processes are far less than those produced by anthropogenic activities [60,61]. Moreover, benzo[a]pyrene produced from natural processes is easily adsorbed by soil organic carbon, and cannot easily migrate from the soil into the groundwater. Former studies have also shown that anthropogenic activities are the main sources of benzo[a]pyrene in the environmental medium [62,63,64,65,66,67]. Given that is shows the highest rates of detection and being over the limit, as well as its high toxicity, benzo[a]pyrene in groundwater achieved the highest *PvOPBT* score in the study area.

In Bole City, Kuitun City, Shawan City, Changji City, Urumqi City and Fukang City in the southern areas of the Junggar Basin, all the groundwater samples with detected benzo[a]pyrene were distributed in industrial parks and/or plants, based on the the distribution of PCOPs (Figure 6) and the survey data on priority pollution sources [46]. The discharge of waste from these industrial activities (e.g., coking gas production, use of industrial boilers, petroleum processing and steel smelting) was the primary source of groundwater benzo[a]pyrene [68]. In addition, frequent transportation in industrial parks and/or plants, tire road-wear and the running of car engines could produce benzo[a]pyrene-containing particles that may enter groundwater through precipitation infiltration, and cause groundwater pollution [69,70,71]. Therefore, the presence of benzo[a]pyrene in groundwater can be attributed to frequent industrial activities and transportation in these areas.

The benzo[a]pyrene in the groundwater samples was detected in the irrigation area (in Jinghe County) and near gas stations (<100 m) (in Hoboksar Mongol Autonomous County, Hefeng County for short). Since benzo[a]pyrene is the main organic component of nitrogen–phosphorus–potassium (NPK) fertilizers, the widespread use of NPK fertilizers may cause benzo[a]pyrene to enter groundwater via the infiltration of polluted irrigation water [72]. Furthermore, the harvesting of cereals and cotton, the major crops in the Junggar Basin, could produce by-products (e.g., straw). This straw is usually incinerated directly on cultivated land or used as a fuel, during which the benzo[a]pyrene-containing residues that are produced could enter the groundwater with rain water, melting water, and irrigation water [73,74]. Besides this, the main components of petroleum and diesel are alkanes, olefins and aromatic hydrocarbons, and benzo[a]pyrene is an important component of aromatic hydrocarbons. Once fuel leakage at gas station occurs, benzo[a]pyrene can enter the groundwater [75]. Therefore, the benzo[a]pyrene in groundwater may be affected by agricultural activities and fuel oil leakage in these areas.

However, all the groundwater samples containing benzo[a]pyrene were distributed in the irrigation area (without industrial activities in the upstream area) in Manas County and Hutubi County in the south, Karamay City in the west and Habahe County, Burqin County, Altay City and Fuhai County in the north. There are no industrial activities in the upstream regions of these areas. Therefore, NPK fertilizer application and straw incineration during agricultural activities were considered the major sources of benzo[a]pyrene in groundwater. The benzo[a]pyrene in the groundwater in Fuyun County was mainly observed in the irrigation area, in plants and in passenger stations, which may have multiple sources, including NPK fertilizer application, straw incineration, industrial activities, tire road-wear and traffic emissions.

Since grains and cottons are the most staple crops in the irrigation area of the Junggar Basin, long-term irrigation with benzo[a]pyrene-containing groundwater could result in the inhibition of crop growth, reductions in crop yield and the accumulation of benzo[a]pyrene in crops. As for humans, contact with and/or intake of benzo[a]pyrene could potentially threaten the nervous system and reproductive system, and cause cancer [63,76,77,78].

### 4.2. 1,2-Dichloroethane

Without natural sources, 1,2-dichloroethane is primarily derived from industrial production (e.g., the manufacture of asphalt, plastic and chemical fibers, printing and petroleum processing) and the raw materials (e.g., pesticides, soil disinfectants and grain fumigants) used in agriculture activities [79,80]. As one of the DNAPLs, 1,2-dichloroethane, which is poorly soluble in water and volatile, can easily enter into the atmosphere and/or infiltrate the vadose zone. However, due to its slow volatilization in the vadose zone, 1,2-dichloroethane in the vadose zone tends to migrate downward into groundwater and subsequently move downstream with the groundwater flow, causing persistent organic pollution [81]. As for humans and animals, long-term contact with and/or intake of 1,2-dichloroethane-containing groundwater could cause cranial nerve injury, gene mutations and cancer [82,83]. Once 1,2-dichloroethane-containing groundwater enters into and accumulates in plants/crops, the photosynthesis and growth of the plants/crops will be inhibited [84].

Groundwater containing 1,2-dichloroethane (accounting for 79% of total groundwater 1,2-dichloroethane samples) was mainly concentrated in industrial parks. Due to the wide distribution of industrial parks in the southern region of the Junggar Basin (37% of the total number of industrial parks in Xinjiang), groundwater in this area is prone to becoming polluted with industrial waste. 1,2-dichloroethane-containing groundwater samples were mainly distributed near industrial parks (<2.0 km) in Kuitun City, Hutubi County, Changji City and Fukang City in the south, in the western Beitun City, and in Fuyun County in the north (Figure 6). 1,2-dichloroethane is widely used in chemical production processes with an annual output reaching up to tens of millions of tons, including metal degreasing, paint removal, adhesive and disinfectant manufacturing, wax and rubber processing, eluent, drug and plastic product synthesis, and vinyl chloride and other chlorinated hydrocarbons, amines and fluorocarbons production. Accordingly, 1,2-dichloroethane-containing wastewater has become common in the petrochemical industry [85]. Therefore, the discharge of industrial wastes (mainly from chemical fiber plants, plastic-manufacturing plants and oil-processing plants in industrial parks) into groundwater is the primary source of 1,2-dichloroethane in groundwater in these areas [46]. on the other hand, groundwater containing 1,2-dichloroethane in Shawan County, Manas County and Jimsar County in the south and Beitun City in the north was mainly distributed in irrigation area, where corn and cotton are the main crops. Wastes from agricultural activities (e.g., soil disinfectant and grain fumigant used in and before the cropping season) were the major sources of 1,2-dichloroethane in groundwater.

### 4.3. Trichloromethane

Trichloromethane is commonly applied in organic synthesis, e.g., the production of CFCs, dyes, anesthetics, antibiotics, flavorings, and solvents/extractants for grease and rubber [86,87,88]. As for humans and animals, contact with and/or intake of trichloromethane could cause gastrointestinal tract damage, hepatotoxicity, dermatitis and related forms of cancer [89]. When accumulated in plants/crops, trichloromethane could inhibit growth [90].

In the study area, 60% of the total groundwater samples containing trichloromethane were distributed in/near (≤1.0 km) industrial parks (including a dye factory, an oil and grease factory, a spice factory and a rubber factory, a water treatment plant and hospitals) in Urumqi City. Due to its high solubility and extraction rate in relation to many organic solvents, trichloromethane is widely applied in industrial production activities (e.g., the production of dyes, grease, fragrances and rubber). Emulsification and entrapment during extraction can produce wastewater containing trichloromethane. Industrial wastewater containing compounds with methyl ketone structures, or acetaldehyde and its halogen derivatives (or substances that can be oxidized to these compounds or derivatives), is usually treated with chloride. This could result in the formation of trichloromethane via the Liben reaction, especially under alkaline conditions [91,92]. Moreover, chlorine and calcium hypochlorite are commonly used in water purification in water treatment plants. Trichloromethane-containing by-products, produced via reactions between chlorine/calcium hypochlorite and natural humus in water, could enter the groundwater via pipeline leakages [93]. Furthermore, medical waste leakage (e.g., anesthetic, antibiotic and disinfection by-products) results from improper waste disposal in medical activities, and this could also lead to the migration of trichloromethane into groundwater.

Other groundwater samples containing trichloromethane were distributed in industrial parks (including a dyestuff plant, an oil and grease plant, a spice factory, a rubber factory, etc.) in Shawan City, Wujiaqu City, Hefeng County and Fuyun County, and these are primarily attributed to the migration of spent liquor from industrial activities. Trichloromethane, which is poorly soluble in water and difficult to biodegrade, has good solubility and mobility in soil [94]. It may easily migrate from the soil and accumulate in groundwater, causing groundwater trichloromethane contamination under proper conditions (e.g., coarse particle vadose deposits and high groundwater level in the groundwater catchment area) [95,96,97].

### 4.4. DDTs

As one of the most effective pesticides, DDTs have been widely used in pest control over the past few decades [98]. The consumption of DDTs in China accounts for 20% of the global consumption [99]. Although DDTs have been prohibited for production and use in since in China [100,101] due to their strong hydrophobicity, high toxicity and recalcitrance, they still pose a serious threat to ecosystems and human health, with wide distribution in various environmental mediums at present [102]. Despite the low detection rate (0.4%), the *PvOPBT* score of DDTs in groundwater was found to be high because of its high toxicity and high concentration. The DDTs in groundwater were found primarily distributed in Beitun City and Jinghe County in the north, where cotton and maize are the major crops. DDTs could inhibit the growth of cotton balls and lead to a drop in cotton yield [103]. Once agricultural products contain residues and accumulations of DDTs enter the human body via food intake, they can cause dysneuria, reproductive system disorders and cancer [104,105,106].

Comparing the scores of each criterion for priority control pollutants shows that the concentration contributes the most to the total score, indicating the importance of controlling organic pollutant concentration in the study area for the screening of PCOPs.

### 4.5. Risk Assessment

The results of the ecological risk assessment indicate that benzo[a]pyrene and DDTs pose a high ecological risk, and that 1,2-dichloroethane poses a medium ecological risk because of the high concentration and high species sensitivity of these PCOPs. Trichloromethane posed no ecological risk and low ecological risk. As one of the key controlling factors in pollutant migration, the lithology of the vadose zone could determine the degree of pollutant migration/blockage (e.g., degradation, adsorption and precipitation). The lager the particle size and the higher the permeability of the vadose zone lithology, the easier it is for the pollutant to migrate into the aquifer. The lithology of the vadose zone in medium–high-ecological risk areas in the southern region of the basin was dominated by sandy loam, whilst that in the north part of the basin was dominated by sand gravel and silty–fine sand. Therefore, it contributed to the downward migration of these PCOPs, resulting in elevated concentrations and detection rates of PCOPs in groundwater [107]. On the other hand, the infiltration recharge of polluted river water could also lead to groundwater pollution. Spatially, 60% of the groundwater PCOPs posing medium–high ecological risk were distributed near rivers (<5 km) in the basin (benzo[a]pyrene and trichloromethane in the Manas River’s waters, for instance) [108]. The distribution of PCOPs in these rivers was similar to that in groundwater. The infiltration recharge of PCOPs-containing river water, therefore, was considered as another source of groundwater PCOPs. Thus, the ecological risk posed by surface water pollution coupled with the growth of animals and vegetation should be of concern, as should the timely identification and remediation of polluted/destroyed ecological environments.

The results of the health risk assessment show that most of the PCOPs in the groundwater samples (58.3%) posed low potential carcinogenic risk (10^−6^ < R < 10^−4^) to human health, and that the benzo[a]pyrene present at 2.6% in groundwater samples posed a high potential carcinogenic risk (R > 10^−4^). The potential carcinogenic risk of these PCOPs for children was 2.1 times that for adults. Due to their lower body weight, pollutant accumulation per unit weight in a child is greater than that in an adult. Accordingly, the health of a child is more sensitive to pollutants than that of an adult [109,110]. Restricted by local infrastructure and economic conditions, reducing exposure to high-carcinogenic risk groundwater and its threats to human health could be a long-term process. In areas with groundwater PCOPs that pose a carcinogenic risk, relevant government departments should take prompt action towards pollution source identification, the prevention of further pollution, pollutant diffusion and practical pollution remediation.

In terms of groundwater source planning in high-carcinogenic risk areas, the investigation of groundwater quality should be carried out in advance to avoid polluted area. Accordingly, the delineation of a well head protection area is required to prevent the direct pollution of groundwater via anthropogenic activities. In terms of groundwater source planning at areas downstream from pollution sources, the concentrations of major pollutants in groundwater should be reduced to meet the quality standard requirements of Class III groundwater (GBT 14848-2017 Groundwater Quality Standard) in China. Besides this, groundwater quality monitoring with higher frequency and more monitoring indices is necessary to ensure timely response to sudden water source pollution events. In addition, relevant government departments should popularize education on drinking water safety, and forbid residents to dig wells or use untreated groundwater in potentially polluted areas.

## 5. Conclusions

In this study, a prioritization method based on *Pv*, *O* and *PBT* scores was applied to identify groundwater PCOPs in the Junggar Basin with the synthetic consideration of the toxicity, distribution and degree of organic pollutants. According to the *PvOPBT* score, four types of groundwater organic pollutants of high priority were identified as PCOPs in the Junggar Basin, including polycyclic-aromatic hydrocarbons, mono-aromatic hydrocarbons, halogenated aliphatic hydrocarbons and organochlorine pesticides. The points of detection of PCOPs in groundwater were concentrated in the middle of the southern region and the northern part of the basin, where anthropogenic activities were intense. Benzo[a]pyrene and DDTs posed high potential ecological risks, while 1,2-dichloroethane and chloroform posed medium and the lowest potential ecological risks, respectively. Among all the PCOPs, the potential health risks of benzo[a]pyrene to human health were the highest. Moreover, the potential health risk for a child was higher than that for an adult. Our findings provide more accurate information on groundwater organic pollutants in the Junggar Basin, which is essential for establishing a list of groundwater PCOPs, and this could help to restrict the use of groundwater PCOPs and could scientifically support the development of relevant guidelines and regulations for environmental monitoring.

Furthermore, two questions remain to be investigated in future studies: (1) the specific sources of PCOPs need further analysis based on a follow-up investigation of the status and distribution of pollutants in the groundwater–surface water–soil system, and (2) in addition to ingestion exposure, health risks related to inhalation exposure and dermal exposure need to be further studies via multi-factor analyses.

## Figures and Tables

**Table 1:** Statistics of organic pollutants in groundwater from the Junggar basin.

Organic Pollutants	CAS ^a^	Sample Quantity	Positive Detection Quantity	Detection Limit (μg/L)	Organic Pollutants	CAS ^a^	Sample Quantity	Positive Detection Quantity	Detection Limit (μg/L)
Benzene	71-43-2	454	1	0.3	Chlorodibromomethane	124-48-1	455	3	0.15
Ethylbenzene	100-41-4	454	1	0.3	Chloroethylene	75-01-4	455	2	0.5
Toluene	108-88-3	454	2	0.3	1,1-Dichloroethylene	75-35-4	455	3	0.2
Bromodichloromethane	75-27-4	455	3	0.15	Bromoform	75-25-2	454	0	0.5
Dichloromethane	75-09-2	454	0	0.5	1,2-Dichloroethylene	540-59-0	455	9	0.2
Trichloroethylene	79-01-6	454	0	0.2	Styrene	100-42-5	454	0	0.25
1,2-Dichloroethane	107-06-2	454	16	0.25	1,2-Dichlorobenzene	95-50-1	454	0	0.1
Chloroform	67-66-3	454	10	0.2	Chlorobenzene	108-90-7	454	0	0.05
Tetrachloroethylene	127-18-4	454	1	0.2	1,3-Dichlorobenzene	541-73-1	454	0	0.1
1,1,1-Trichloroethane	2747-58-2	454	0	0.15	1,4-Dichlorobenzene	106-46-7	454	0	0.1
Carbon Tetrachloride	56-23-5	454	1	0.2	β-Hexachlorocyclohexane	319-85-7	453	5	0.01
1,2-Dichloropropane	78-87-5	455	3	0.15	α-Hexachlorocyclohexane	319-84-6	452	2	0.01
1,1,2-Trichloroethane	79-00-5	454	1	0.15	δ-Hexachlorocyclohexane	319-86-8	454	2	0.01
Benzo[a]pyrene	50-32-8	453	38	0.002	Lindane	58-89-9	452	3	0.5
Dichlorodiphenyltric-hloroethanes(DDTs)	50-29-3	454	2	0.01	1,3-Dimethylbenzene	108-38-3	454	1	0.5
Hexachlorobenzene	118-74-1	453	0	0.005	1,4-Dimethylbenzene	106-42-3	454	1	0.5
1,2,4-Trichlorobenzene	120-82-1	454	0	0.2	1,2-Dimethylbenzene	95-47-6	454	1	0.3

^a^ Chemical abstracts service number.

## Data Availability

The datasets used and/or analyzed during the current study are available from the corresponding author on reasonable request.
